# Identification and masking of artifactual and misleading within-host variants in deep-sequencing SARS-CoV-2 data

**DOI:** 10.1093/molbev/msag209

**Published:** 2026-08-18

**Authors:** Klara Marie Anker, Rosario Evans Pena, Steven A Kemp, Joseph Clarke, Lele Zhao, David Bonsall, Nicholas Grayson, Matthew Bashton, Ann Sarah Walker, Tanya Golubchik, Matthew Hall, Katrina Lythgoe

**Affiliations:** Pandemic Sciences Institute, University of Oxford, Oxford, UK; Big Data Institute, Nuffield Department of Medicine, University of Oxford, Oxford, UK; Pandemic Sciences Institute, University of Oxford, Oxford, UK; Department of Biology, University of Oxford, Oxford, UK; Pandemic Sciences Institute, University of Oxford, Oxford, UK; Big Data Institute, Nuffield Department of Medicine, University of Oxford, Oxford, UK; Big Data Institute, Nuffield Department of Medicine, University of Oxford, Oxford, UK; Pandemic Sciences Institute, University of Oxford, Oxford, UK; Big Data Institute, Nuffield Department of Medicine, University of Oxford, Oxford, UK; Pandemic Sciences Institute, University of Oxford, Oxford, UK; Big Data Institute, Nuffield Department of Medicine, University of Oxford, Oxford, UK; Wellcome Centre for Human Genetics, Nuffield Department of Medicine, NIHR Biomedical Research Centre, University of Oxford, Oxford, UK; Pandemic Sciences Institute, University of Oxford, Oxford, UK; Big Data Institute, Nuffield Department of Medicine, University of Oxford, Oxford, UK; Wellcome Centre for Human Genetics, Nuffield Department of Medicine, NIHR Biomedical Research Centre, University of Oxford, Oxford, UK; Hub for Biotechnology in the Built Environment, Northumbria University, Newcastle-Upon-Tyne, UK; Nuffield Department of Medicine, University of Oxford, Oxford, UK; The National Institute for Health Research Health Protection Research Unit in Healthcare Associated Infections and Antimicrobial Resistance, University of Oxford, Oxford, UK; The National Institute for Health Research Oxford Biomedical Research Centre, University of Oxford, Oxford, UK; Big Data Institute, Nuffield Department of Medicine, University of Oxford, Oxford, UK; The Sydney Infectious Diseases Institute (Sydney ID), School of Medical Sciences, University of Sydney, Sydney, Australia; Pandemic Sciences Institute, University of Oxford, Oxford, UK; Big Data Institute, Nuffield Department of Medicine, University of Oxford, Oxford, UK; Department of Clinical Research, London School of Hygiene & Tropical Medicine, London, UK; Pandemic Sciences Institute, University of Oxford, Oxford, UK; Big Data Institute, Nuffield Department of Medicine, University of Oxford, Oxford, UK; Department of Biology, University of Oxford, Oxford, UK

**Keywords:** intrahost variants, sequencing artifacts, variant masking, transmission bottleneck, viral evolution, SARS-CoV-2

## Abstract

Deep-sequencing data are increasingly used to study within-host viral diversity and to inform evolutionary inference. For SARS-CoV-2, analyses based on intra-host single-nucleotide variants (iSNVs) have been widely applied to quantify within-host diversity and infer transmission dynamics. However, these applications critically depend on the reliable identification of low-frequency variants, which remain vulnerable to systematic and technical artifacts. In this study, we show that recurrent artifactual iSNVs are common in large-scale SARS-CoV-2 sequencing data and can persist even under conservative minor allele frequency thresholds. Using data from the UK's Office for National Statistics COVID-19 Infection Survey, we demonstrate that such artifacts are predominantly sequencing center-specific rather than primer-specific. Each center exhibits a modest, distinct set of recurrent artifactual variants showing little overlap with sites routinely masked at the consensus level. To address this, we developed a systematic, dataset-aware framework that uses recurrence within sequencing datasets to identify small, noise-adapted sets of artifactual iSNVs to mask. Applying this framework reduces spurious sharing of low-frequency variants between samples and qualitatively alters downstream inferences, including estimates of within-host diversity and transmission bottleneck sizes. Although this study focused on SARS-CoV-2, it is likely that recurrent artifactual iSNVs will be problematic for other viruses as mass-sequencing becomes increasingly routine. Together, these findings highlight the importance of explicit, dataset-aware artifact control for robust inference from within-host variation, particularly as genomic studies increasingly seek to exploit sub-consensus diversity in rapidly evolving pathogens.

## Introduction

The global emergence of SARS-CoV-2 and the evolution of multiple variants of concern have demonstrated the critical role of genomic surveillance in managing the pandemic through the use of whole-genome sequencing (WGS) (Zhou et al. 2020). WGS captures the genetic diversity of a viral population within a sample, with the consensus sequence essentially representing the most common allele at each position in the genome. It is the consensus sequence that is typically used for between-host phylogenetic analyses. Analysis of intra-host single-nucleotide variants (iSNVs), on the other hand, is typically used to track the evolution of the virus within-host and to establish, for example, how many viral particles are transmitted at infection.

Despite its benefits, the application of WGS in tracking SARS-CoV-2 suffers from inconsistencies, likely due to varied sequencing methodologies, sample and analysis pipelines, and laboratory practices (Chiara et al. 2020; Stoler and Nekrutenko 2021; Berno et al. 2022; Mostefai et al. 2024). Here, we refer to observed viral variants that appear in sequencing data but are not present in sampled virus populations as “artifacts”. At the consensus level, known artifacts, which were first noticed as they appeared as common homoplasies on phylogenies (De Maio et al. 2020; Hunt et al. 2026), are often primer-specific (Ellegaard et al. 2025) or lab-specific (Turakhia et al. 2020), but could have various causes, including sequence alignment errors (Ellegaard et al. 2025) and changes introduced into the genome due to sample storage and preparation.

Additionally, artifacts have been observed at the within-host level. When examining within-host diversity, it is typical to exclude within-host variants with a minor allele frequency (MAF) of less than 2% to 5% (Poon et al. 2016; McCrone et al. 2018; Lythgoe et al. 2021). Nevertheless, variants that are generally present at low frequencies within hosts but recur across a large proportion of samples have been reported (Lythgoe et al. 2021; Martin and Koelle 2021; Tonkin-Hill et al. 2021; Mostefai et al. 2024). It is unlikely that these highly shared iSNVs represent heritable genetic variation, given the short duration of infection and the typically tight transmission bottlenecks reported for SARS-CoV-2, and we therefore refer to them as artifactual iSNVs (McCrone and Lauring 2016; Lythgoe et al. 2021; Li et al. 2022). Crucially, failing to mask positions in the genome where artifactual iSNVs are common can lead to erroneous conclusions about within-host genetic diversity, evolution, and transmission (Lythgoe et al. 2021; Martin and Koelle 2021).

Although the presence of artifactual iSNVs can be reduced through careful experimental design and replicate sampling (Roder et al. 2023; Walter et al. 2023; Bendall et al. 2025), the vast majority of SARS-CoV-2 sequences were generated primarily to obtain consensus genomes for phylogenetic surveillance and to track the emergence of antigenically distinct variants of concern. Examining the within-host diversity of these expensive-to-generate sequenced samples will allow information from this massive collection of sequences to be maximized, but to do so, it is essential to develop approaches that allow quality data to be extracted while discarding artifacts.

To gain a clearer picture of the prevalence of artifactual iSNVs in SARS-CoV-2 data, we analyzed more than 123,000 whole-genome sequences collected as part of the United Kingdom's Office for National Statistics Coronavirus (COVID-19) Infection Survey (ONS-CIS) (Pouwels et al. 2021; Lythgoe et al. 2023). We investigated different strategies for identifying and masking iSNVs that recur across a large proportion of samples. Regardless of the strategy used, we observed a large number of highly shared artifactual iSNVs in the data, many of which were sequencing center-specific. We demonstrate that failing to mask artifactual sites can lead to erroneous conclusions in downstream analyses, including considerably overestimating levels of within-host diversity and the size of the transmission bottleneck. We therefore advocate that all studies that utilize SARS-CoV-2 within-host diversity should make efforts to identify and mask highly shared iSNVs and should carefully account for both noise and technical artifacts inherent to viral deep-sequencing data. Moreover, researchers should remain vigilant to the possible existence of highly shared artifactual variants in other pathogen-host systems, including disease X.

## Results

### Sequencing center differences in iSNV abundance and sharing among samples

The ONS-CIS initially ran from 2020 April 27 to 2023 March 13 before being paused and then closed. We identified a total of 123,233 high-quality sequences from samples that were collected as part of the ONS-CIS, which met the criteria of a read depth ≥10× across ≥50% of positions in the genome. These sequences were generated by several laboratories throughout the course of the study using either a bait-capture approach (ve-SEQ) (Bonsall et al. 2020) or the ARTIC amplicon-based approach with primer versions 3, 4, or 4.1 (Quick 2020) (Table 1, Figure S1). Hereafter, we refer to the combination of sequencing approach and primer version as the method.

**Table 1:** Sequencing laboratories and methods.

Sequencing lab	Short code	Method	Library preparation kit	Sequencing platform	Sequencing center-method	No. of high-quality genomes
Wellcome Sanger Institute (SANG)	MILK	ARTIC v3	NEB Ultra II	Illumina NovaSeq	SANG ARTIC 3	285
	QEUH	ARTIC v3			SANG ARTIC 3	172
		ARTIC v4.1			SANG ARTIC 4.1	64,010
	BRBR	ARTIC v4.1			SANG ARTIC 4.1	161
	LSPA	ARTIC v4.1			SANG ARTIC 4.1	2,126
Northumbria University	NORT	ARTIC v3	Illumina DNA Prep^b^	Illumina NextSeq 550	NORT ARTIC 3	8,754
		ARTIC v4			NORT ARTIC 4	25,975
		ARTIC v4.1			NORT ARTIC 4.1	11,893
Quadram Institute Bioscience	NORW	ARTIC v?^a^	Nextera^b^	Illumina NextSeq 500	NORW ARTIC?	900
Oxford Viromics, NDM, University of Oxford	OXON	ve-SEQ	SMARTer Pico v2	Illumina NovaSeq	OXON ve-SEQ	6,608
Respiratory Virus Unit, Microbiology Services Colindale, Public Health England	PHEC	ARTIC v3	Nextera XT	Illumina NextSeq 550	PHEC ARTIC 3	2,349^c^

Samples were initially handled at different originating laboratories and then sent to other laboratories for sequencing as indicated by the Sequencing lab column. The short codes refer to the COG-UK prefixes found in the sequence names for each sample. The Sequencing center-method column specifies the categorization of samples used throughout this paper, based on the sequencing laboratory, sequencing approach, and primer version used. The number of high-quality sequences (depth of ≥10× at ≥50% of genome positions) obtained from each of the combinations is shown in the last column.

^a^ARTIC primer version is unknown. Based on timings, we expect v4 or v4.1 was used.

^b^The Illumina DNA prep was previously called Nextera DNA Flex, and “Nextera” might refer to this kit. NORW performed overflow sequencing for NORT, and both used the same end-to-end protocol, CoronaHiT (Baker et al. 2021). NORW sequences were not prepared in NORT, nor vice versa.

^c^2,073 extra samples had no information on primer version and were excluded from this analysis.

To explore intra-host diversity within this dataset, we applied a range of minimum MAF thresholds from 2% to 20% required to call iSNVs, restricting the analysis to sites with at least 1,000× total read coverage. Initially, we also tested alternative read depth thresholds of 10× and 100× (Figure S2); however, varying this threshold had minimal effect compared to varying the MAF threshold for calling iSNVs, and we decided to use the strict 1,000× depth threshold for subsequent analysis.

**Figure 1:**
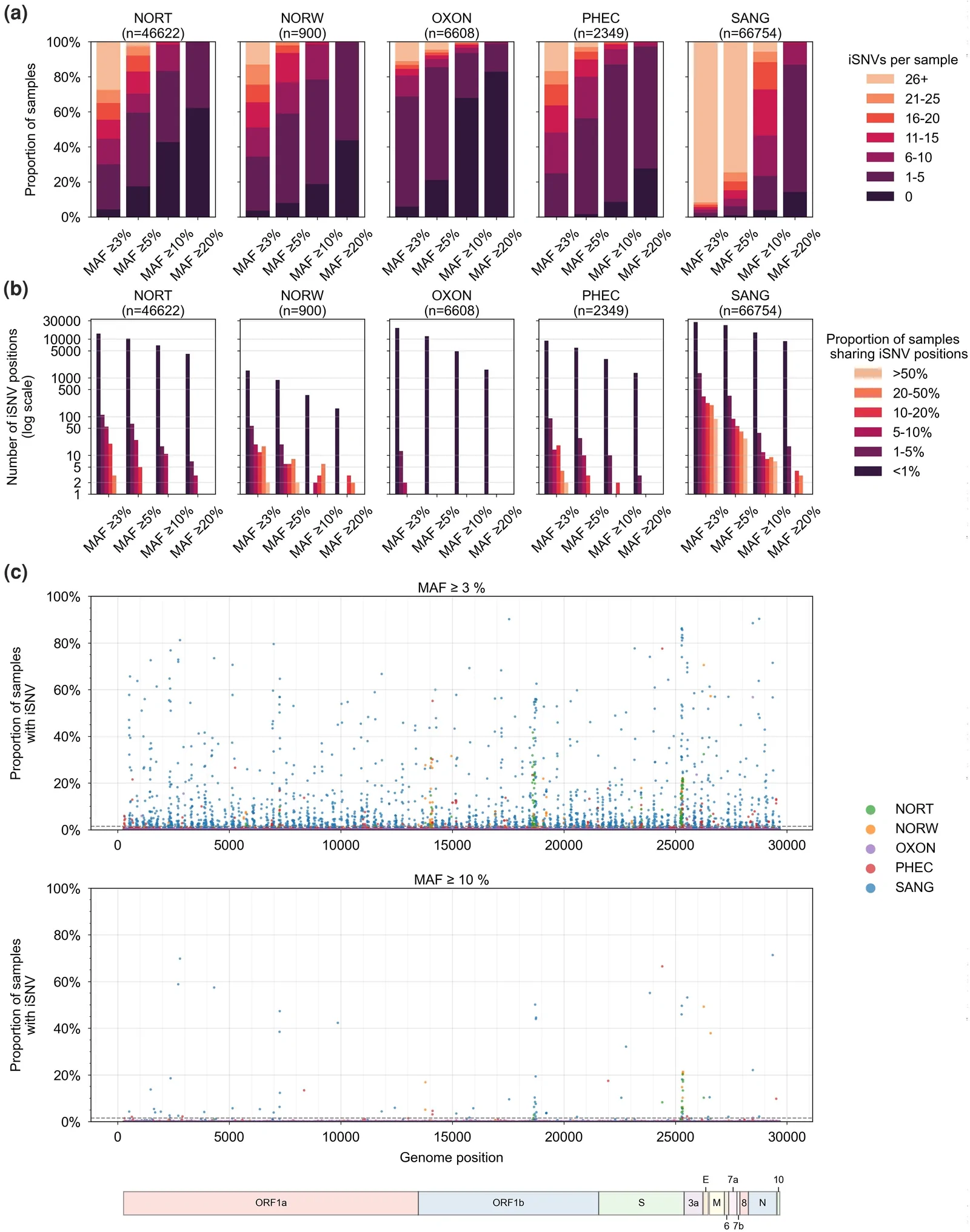
Distribution of iSNVs across samples processed at multiple sequencing centers, demonstrating widespread occurrence and high prevalence. a) Stacked bar plots showing the proportion of samples carrying different numbers of iSNVs per sample when using various MAF thresholds (3%, 5%, 10%, or 20%) to call the iSNVs. Each stacked bar segment represents a specific range of iSNV counts per sample. At lower MAF thresholds, samples have generally more iSNVs, whereas at higher thresholds, most samples contain few or zero iSNVs. b) Log10-scaled bar plots show, for different MAF thresholds, the number of distinct genomic positions that contain iSNVs in various proportions of samples. For each MAF threshold, bar segments represent different proportions of samples sharing the number of iSNV positions shown on the *y* axis. Most iSNV positions are shared in only a low proportion of samples, but in some sequencing centers, some positions are shared by more than half of the samples, even at higher MAF thresholds. c) Genome-wide map of iSNV prevalence. Each dot represents a nucleotide position (*x* axis) and the percentage of samples (*y* axis) that contain an iSNV at this position, with sequencing centers indicated in the legend. The top panel shows iSNVs at MAF ≥3%, and the bottom panel shows iSNVs at MAF ≥10%. A gray, dashed line indicates a 1.5% proportion of samples. A schematic at the bottom shows the SARS-CoV-2 coding regions relative to the genomic positions.

Increasing the MAF threshold generally reduced the number of observed variants per sample, but even at the same MAF thresholds, the number of observed iSNVs per sample differed markedly between sequencing centers (Fig. 1, Figure S2). We emphasize that some of these sequencing centers were processing extremely high volumes of samples, with a priority on generating consensus genomes, not producing data for sub-consensus analysis. Therefore, it is unsurprising that these data exhibit greater sub-consensus noise and variation than would be expected from pipelines specifically optimized for minor variant detection. We also considered the genomic positions at which iSNVs were observed across samples. As expected, the total number of positions where iSNVs were observed decreased as MAF thresholds increased, but we also found that some positions were observed in a high proportion of samples from the same sequencing center, even when using high MAF thresholds (Fig. 1b). While the vast majority of iSNVs were observed in fewer than 1% of samples at any sequencing center and MAF threshold, some centers exhibited numerous iSNV positions that were highly recurrent, appearing in as many as 20% to 50% of samples, particularly at the lower MAF thresholds of 3% to 5% (Fig. 1b, Figure S3b).

Highly shared iSNVs seemed to be sequencing center-specific, with distinct patterns in their genomic distribution across datasets. In the NORT and NORW data, highly shared iSNVs were largely concentrated within narrow genomic regions, with several positions remaining detectable even at high MAF thresholds (Fig. 1c). It is worth noting here that NORW sequenced samples when NORT exceeded capacity, and although samples were prepared entirely independently, they did use the same end-to-end protocol, CoronaHit (Baker et al. 2021). In contrast, SANG exhibited a more even, periodic distribution of highly shared iSNVs along the genome. Although many recurrent iSNVs were most apparent at lower MAF thresholds, others remained highly prevalent even when MAF thresholds were increased. Notably, clusters of highly shared variants were consistently observed around positions ∼18,600 and ∼25,200 that were shared by up to half of all samples from NORT, NORW, and SANG sequencing centers, and these signals persisted even at MAF thresholds ≥10% (Fig. 1c). In all sequencing centers, the distribution of iSNVs across positions was vastly different from the neutral expectation (Table S1), and this, combined with the different patterns among sequencing centers, strongly suggests that most highly shared iSNVs are artifactual.

### Choosing appropriate thresholds to discriminate artifactual iSNVs from true iSNVs

The observation of highly shared, sequencing center-specific iSNVs suggests that many of them are artifactual and should therefore be discarded for downstream analyses. Here, we suggest a relatively straightforward approach for identifying artifactual iSNVs based on setting appropriate thresholds for the minimum MAF needed to call iSNVs and on the proportion of samples in which a particular iSNV is observed.

**Figure 2:**
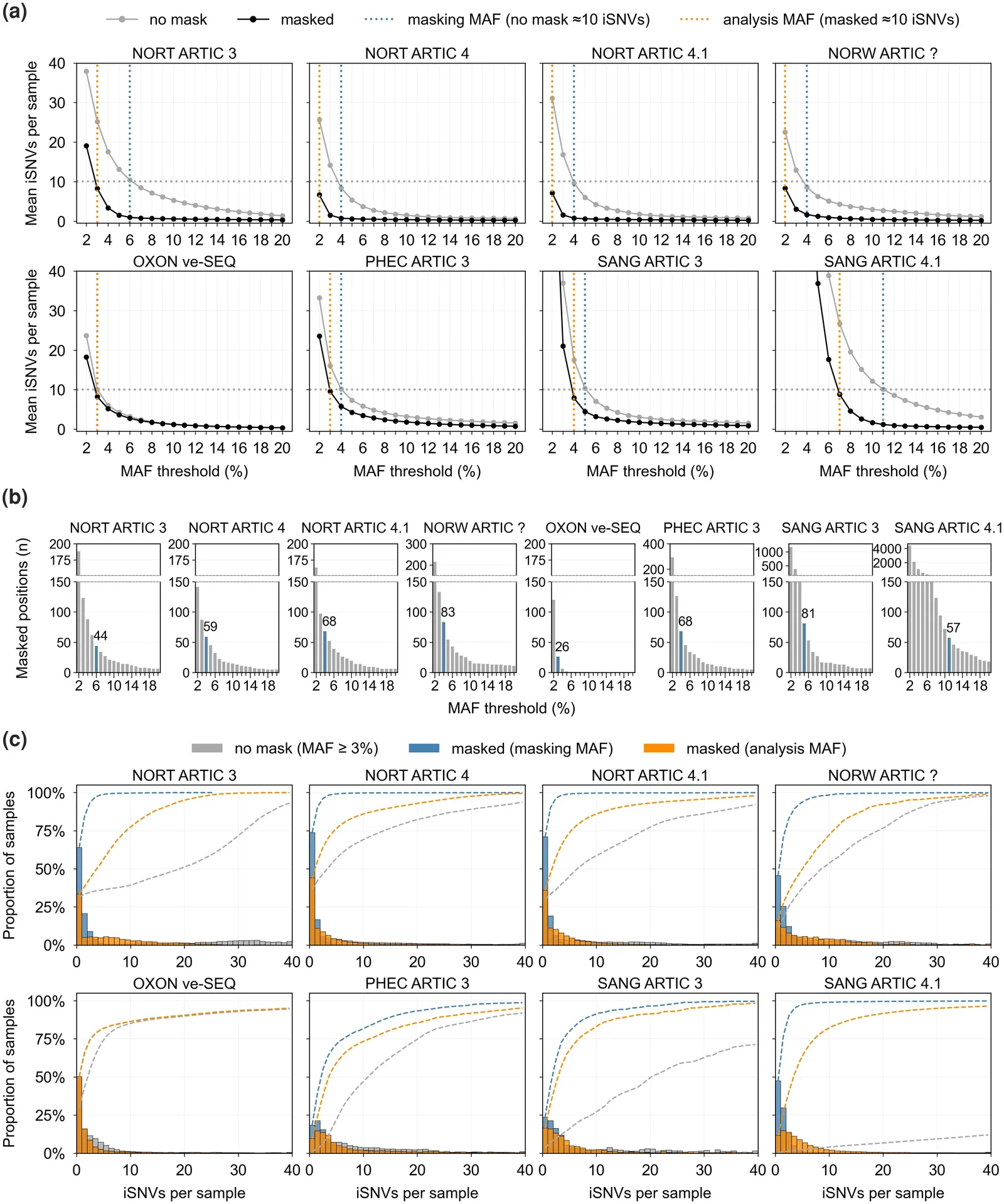
Masking of artifactual iSNVs based on varied MAF thresholds and recurrence between samples. a) Mean number of iSNVs per sample (*y* axis) before and after masking artifactual variants for different analysis MAF thresholds (*x* axis). Two different sets of connected data points show the numbers for unmasked data, and for data after masking positions shared by more than 1.5% of samples at the “masking’ MAF threshold. A horizontal dotted line indicates the “reference” mean number of iSNVs (∼10) and the “masking” MAF threshold is identified as the MAF threshold where the unmasked data is closest to this reference number (blue vertical dotted line). A second vertical dotted line (analysis MAF, orange) indicates the MAF threshold where the masked data is closest to the reference number of iSNVs per sample. b) Number of genomic positions masked (*y* axis) when using various masking MAF thresholds for masking iSNVs that are shared among more than 1.5% of samples. Highlighted bars show the MAF threshold and number of masked positions used in panel (a). Notice that all panels share the same *y* axis scale until 150 masked positions, after which the *y* axis is split to show more varied numbers of masked sites at low MAF thresholds for some sequencing centers. c) Histograms and cumulative distribution functions (CDFs) (dashed lines) showing the proportion of samples from each sequencing center and method (*y* axis) with various total numbers of iSNVs (*x* axis). Different sets of histogram and CDF curves show iSNV counts for either unmasked data analyzed at a 3% MAF threshold (no mask, gray), or for masked data analyzed at the masking MAF threshold (blue), or analysis MAF threshold (orange) identified for the sequencing center and method in panel (a). The *x* axes have been cut at 40 iSNVs per sample, and thus the full distributions are not shown in all panels.

To determine appropriate “masking” MAF thresholds for identifying artifactual iSNVs in the absence of duplicate sequencing, we made the pragmatic assumption that the distribution of observed iSNVs among samples is likely to be similar across sequencing center and method combinations but could be inflated by highly recurrent, technical artifacts. Here, method refers to whether ve-SEQ or ARTIC was used, and if ARTIC was used with primer set v3, v4, or v4.1. Other aspects of the protocols used likely differed between sequencing centers to greater or lesser extents. In a previous study, using a different SARS-CoV-2 dataset and the ve-SEQ protocol (Lythgoe et al. 2021), we used a MAF threshold of 3%, which was supported by duplicate sequencing and sequencing of synthetic genomes. Since the OXON ONS-CIS samples in this study were sequenced in the same sequencing center and using the same protocol, we continued to use the 3% threshold for these sequences, resulting in an average of approximately ten iSNVs per sample before masking artifactual positions (Fig. 2a, horizontal dotted lines). This aligns with variant numbers previously reported by us and others (Lythgoe et al. 2021; Tonkin-Hill et al. 2021; Bendall et al. 2023). We therefore used this number as a reference to identify the MAF thresholds at which the mean number of iSNVs per sample was closest to 10 for each sequencing center-method combination, with resulting MAF thresholds ranging between 3% and 11% (Fig. 2a). To give a specific example, for NORT ARTIC 4, a mean close to 10 iSNVs per sample was observed before masking when using a MAF threshold of 4%, giving a masking MAF threshold of 4% (the point closest to the horizontal dotted line in Fig. 2a).

Using these masking MAF thresholds, we then identified specific iSNVs to mask as artifactual based on how commonly they were shared among samples (Figure S4a). Such a strategy will inevitably require a balance between sensitivity and specificity since true iSNVs may also appear relatively frequently in samples, especially if they are adaptive within the host. In our previous study, we used a threshold of 1.5% of samples sharing the same iSNV position to identify and mask highly shared iSNVs, based on their MAF distributions across samples and sequencing of synthetic genomes (Lythgoe et al. 2021). Using this same 1.5% sharing threshold, and the specific MAF thresholds for each sequencing center-method combination, we identified between 26 and 83 artifactual iSNV positions per combination to mask using what we call the “adaptive” masking scheme (Fig. 2b, Table S2). For example, for NORT ARTIC 4, using the masking MAF threshold of 4% to identify iSNVs, we identified 59 positions in the genome where an iSNV was present in more than 1.5% of samples and which were therefore labeled artifactual positions and subsequently masked.

Even though this resulted in masking less than 0.3% of the SARS-CoV-2 genome, with the exception of OXON, the reduction in the number of observed iSNVs per sample was substantial (Fig. 2a, compare the unmasked and masked lines). This is consistent with a small set of highly recurrent artifactual positions contributing disproportionately to the observed number of iSNVs per sample. To avoid using overly conservative MAF thresholds driven by the presence of artifacts in a high proportion of samples, which might prevent us from identifying genuine low-frequency variants, we next determined “analysis” MAF thresholds for each sequencing center-method combination (Figure S4b). We still used ten iSNVs per sample as the reference, but now, after masking, this resulted in analysis MAF thresholds ranging between 2% and 7% (Fig. 2a, orange vertical dotted lines). Taking our example of NORT ARTIC 4, after masking artifactual positions but retaining the 4% MAF threshold for calling iSNVs, we now observed <2 iSNVs per sample. Reducing the MAF threshold to 2% (the minimum MAF threshold we considered), we were able to recover close to eight iSNVs per sample. This adaptive masking and analysis approach substantially reduced noise-driven variation across centers while also removing suspicious highly shared variants and made the iSNV distributions across samples converge toward similar patterns across the datasets, although noting that the different analysis MAF thresholds used mean they are not directly comparable (Fig. 2c). It is also important to note that the number of iSNVs observed per sample will depend on the number of positions at which an iSNV could be observed (ie ≥1,000× depth) with patterns varying across sequencing centers (Figure S5).

As a sense check that we were identifying a genuine signal, we tested whether the same artifactual positions were identified using the full range of MAF (2% to 20%) and sharing (0.5% to 20%) threshold combinations. Although the resultant sets of positions to mask varied greatly in size, for each sequencing center-method combination, we observed the expected nesting of these sets, with the smaller sets generally contained within the larger ones and reflected by overlap coefficients close to 1 (Figure S4c). This indicates that a consistent group of recurrent artifacts was detected across thresholds. The noisier datasets tended to have lower (but still high) overlap coefficients, highlighting the difficulty of distinguishing between noise, artifacts, and genuine variants. We also note that different MAF and sharing thresholds are likely to pick up different biological or technical processes; a mechanism leading to very low-frequency variants in many samples may well be different from one creating higher-frequency variants in fewer samples.

As a final sense check, we tested the masking of the most frequently flagged positions across all possible masking schemes for each sequencing center-method combination, specifically positions that appeared in >20% of all threshold combinations, calling this the “prevalent mask” scheme (Figure S6). Apart from SANG ARTIC 4.1, this resulted in masking fewer positions, slightly more iSNVs per sample after masking, but similar analysis MAFs. The prevalent mask has the advantage of making no assumptions about the expected number of iSNVs per sample; however, by focusing only on the most frequently flagged positions, it primarily captures the strongest recurrent artifacts and may miss rarer or more context-specific artifacts. Nonetheless, in situations where there is no prior information on the expected number of iSNVs per sample, as is likely to be the case for other viruses or SARS-CoV-2 in nonhuman hosts, this could be a good approach.

### Highly shared iSNVs are laboratory- and method-specific

**Figure 3:**
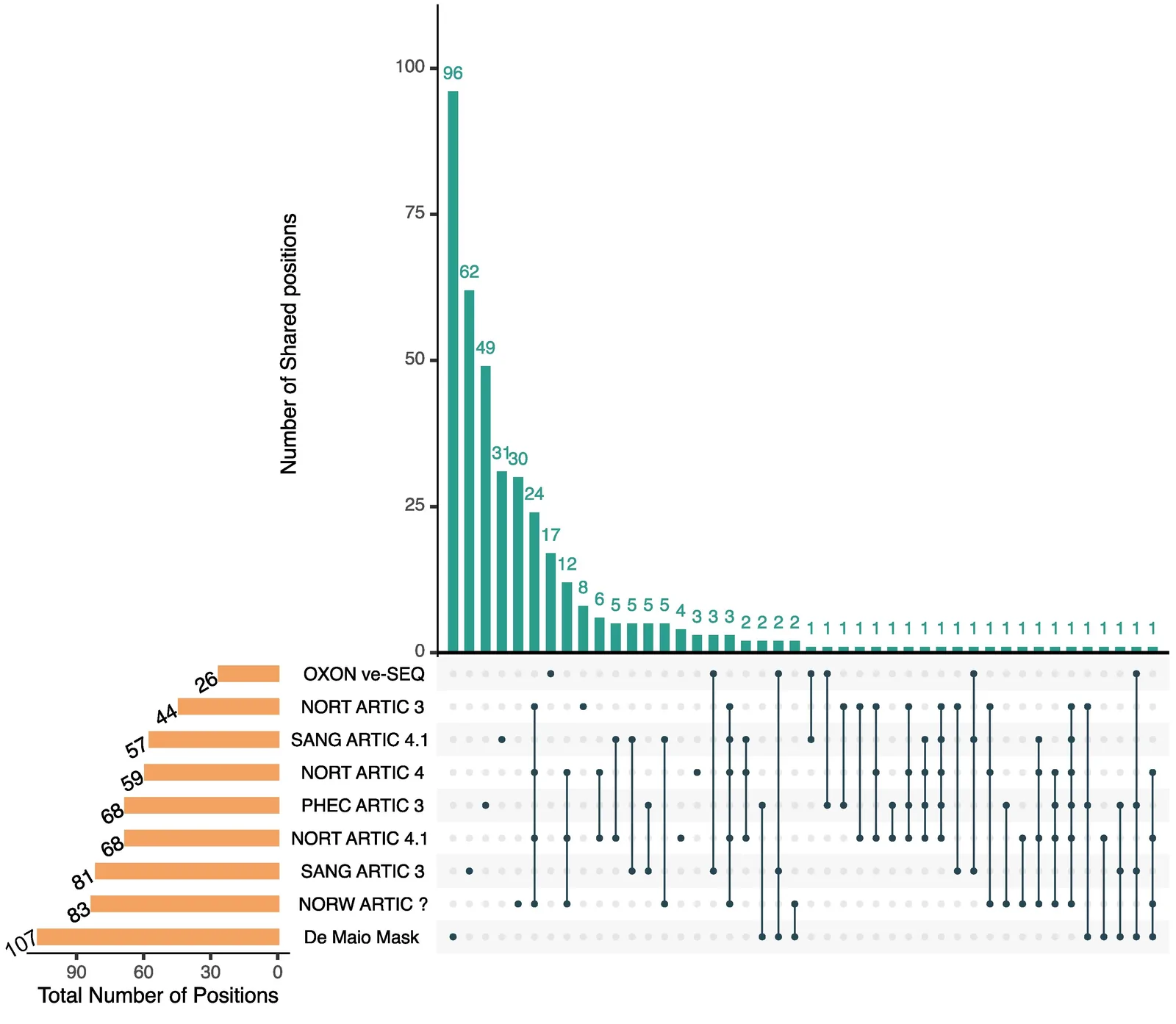
An upset plot showing intersections of masked positions using the adaptive masking strategy. Horizontal bars represent the total number of positions masked for each sequencing center-method combination. The common homoplasy masking set recommended by De Maio et al. (2020) is also shown for comparison. Points in each of the rows indicate sets of positions found within the corresponding masking set, either alone or, if connected to other dots, the number of positions shared between these mask sets given on the left. Vertical bars above the points show the number of positions in the specific intersecting sets or which are unique to one set. Most positions are sequencing center-specific.

While our suggested masking strategies resulted in comparable numbers of masked positions between sequencing centers and methods (Fig. 2b, Table S2), the sets themselves were highly sequencing center-specific and did not reveal artifactual iSNVs that were consistently observed across British SARS-CoV-2 isolates (Fig. 3). Sequencing was not performed regionally, so this cannot explain the differences; moreover, we observed sharp temporal changes in the proportion of samples with iSNVs at artifactual positions that correspond to the switching of sequencing center(s) during the ONS-CIS and which cannot reasonably be explained by biological or epidemiological processes (Figure S7). For most masking sets, approximately 50% to 75% of the masked positions were unique to the given sequencing center and method, and only 11 out of 107 positions overlapped the previously reported list of problematic sites to mask at the consensus level (De Maio et al. 2020). At the NORT sequencing center, many masked positions were shared across the ARTIC v3, v4, and v4.1 primers, indicating that artifacts were predominantly sequencing center rather than primer-specific. The NORT and NORW masks overlapped substantially (45 of 83 NORW masked positions were present in at least one of the NORT masks), which is likely because NORW and NORT used the same protocol, CoronaHit (Baker et al. 2021), for sample preparation and sequencing.

### Considering possible sources of recurrent artifactual iSNVs

To evaluate potential sources of artifactual iSNVs, we examined strand bias, the genomic distribution of iSNVs, their relationship to ARTIC primer-binding regions and amplicon structure, and mutational signatures. Strand bias refers to sequencing artifacts in which reads supporting a variant are unevenly distributed between forward and reverse strands and is used as an indicator of systematic errors in high-throughput sequencing data. In amplicon-based sequencing, such bias can arise from primer mismatches or uneven amplification and is therefore often used as a filter to reduce false-positive variant calls (Guo et al. 2012).

Samples from the OXON sequencing center were excluded from this analysis, as the ve-SEQ protocol is capture-based and not subject to the primer-related strand artifacts associated with ARTIC amplicon sequencing. To test whether strand bias was associated with artifactual iSNVs, we pooled data from 100 randomly selected samples from each sequencing center-method combination (see Methods). For each genomic position, we assessed whether there was a significant excess of forward or reverse reads using Fisher's exact test and examined the overlap between significantly strand-biased positions and the corresponding mask sets. The NORT and PHEC datasets exhibited relatively few strand-biased positions and limited overlap with their respective mask sets (NORT ARTIC v3: 3/44; NORT ARTIC v4: 7/59; PHEC: 9/68), whereas the NORW dataset showed a greater number of strand-biased positions and a higher overlap with the mask set (NORW: 22/68) (Figure S8a and b). In contrast, the SANG datasets contained a large number of strand-biased positions (1,325 in SANG ARTIC v3 and 930 in SANG ARTIC v4.1), with 44 of the 81 masked positions in the SANG ARTIC v3 dataset overlapping strand-biased sites (Figure S8c). Strand bias therefore appears to contribute to some artifactual iSNVs in a center- and method-specific manner, particularly in the SANG datasets. However, because only a subset of masked positions showed strong strand bias, it does not fully explain the artifactual iSNVs identified within each sequencing center-method combination.

Given the previously observed periodic distribution of highly shared iSNVs, particularly in the SANG datasets, we next examined the distance of iSNV positions to primer-binding regions and amplicon ends. This analysis revealed peaks of highly shared iSNVs located approximately 150 to 170 bp from primers and amplicon ends in the SANG datasets (Figure S9). Such spacing is consistent with Illumina end-of-read errors accumulating near the centers of ARTIC amplicons, as previously reported (Dong et al. 2025). Although low-quality bases were filtered out before calculating nucleotide frequencies at each genomic position, the base-quality threshold used was relatively permissive, so some end-of-read errors may have remained and contributed to minor variant calls at these positions. SANG was the only sequencing center to use a non-fragmentation library preparation strategy, whereas the other datasets were generated using protocols that randomly fragment amplicons prior to sequencing, which may contribute to the absence of similar patterns in those datasets.

We also assessed whether the highly shared, masked iSNVs were directly associated with primer-binding regions for each ARTIC primer version and observed only modest overlaps (Figure S8a to c). To formalize this, we tested whether masked positions fell within primer-binding regions more often than expected by chance using a simple hypergeometric model with the full SARS-CoV-2 genome as the background. Under this null, the expected number of overlaps is the fraction of the genome contained in primer-binding regions multiplied by the number of masked positions. Across all three primer versions, observed overlaps were close to random expectation and were not significant after correction for multiple testing (Table S3), indicating that residual primer sequence or incomplete primer trimming is unlikely to be a dominant source of artifactual iSNVs. Nonetheless, a small subset of the artifactual minor alleles was periodically seen at high frequency among global consensus sequences during the relevant sampling periods, as queried using the CoV-Spectrum LAPIS instance (Chen et al. 2022), and almost all of these occurred within primer-binding regions used by the corresponding sequencing protocol (25/26; Figure S10). Most of these alleles also matched the corresponding primer sequence (21/25), supporting a primer-derived origin for this subset of artifacts rather than contamination.

Examination of the genomic distribution of masked positions revealed that, while artifactual iSNVs were broadly dispersed across the genome for most sequencing centers (Figure S11a), NORT and NORW specifically exhibited clusters of masked sites concentrated within discrete genomic intervals, some of which coincided with regions of reduced sequencing coverage (Figure S11b to d). Such patterns are consistent with suboptimal amplification of specific ARTIC amplicons, which would be expected to increase stochastic error rates and promote recurrent artifactual variant calls within affected regions.

Finally, we compared the mutational signatures at artifact versus non-artifact positions for each sequencing center-method combination (Figure S12). If masking, combined with applying the appropriate analysis MAF threshold, removes all false iSNVs while retaining true variants, then we would expect to observe different mutational signatures between the two groups due to the action of different mutational processes. For most sequencing center-method combinations, the mutational signatures of true versus artifactual positions differed markedly, with, for example, transitions more common among true variants compared to artifactual variants, but with G > T mutations (associated with oxidative damage) more common among artifactual variants. There were clear differences in the mutational signatures of artifactual variants among sequencing centers; for instance, OXON had an excess of T > G mutations but few G > T mutations, suggesting the action of different underlying error mechanisms. In some cases, most noticeably SANG ARTIC 4.1, the true and artifactual variants had similar mutational signatures, most likely because, while the masking and threshold approach is successful in removing systematically misleading variants, it does not remove all erroneous iSNVs.

Taken together, these analyses indicate that artifactual iSNVs arise through multiple, dataset-specific technical processes, none of which alone fully explains the observed patterns and thus underscores the need for adaptive, center-specific approaches to identify and mask recurrent artifacts.

### Artifactual positions can inflate measures of within-host diversity and the transmission bottleneck size

Accurate characterization of within-host diversity is essential for understanding within-host viral evolution and inferring transmission dynamics. However, artifactual iSNVs can inflate diversity estimates and create spurious genetic similarities between samples. To assess how such artifacts could affect downstream analyses, we compared iSNV frequencies in related and unrelated samples and estimated transmission bottleneck sizes before and after masking recurrent artifactual positions.

**Figure 4:**
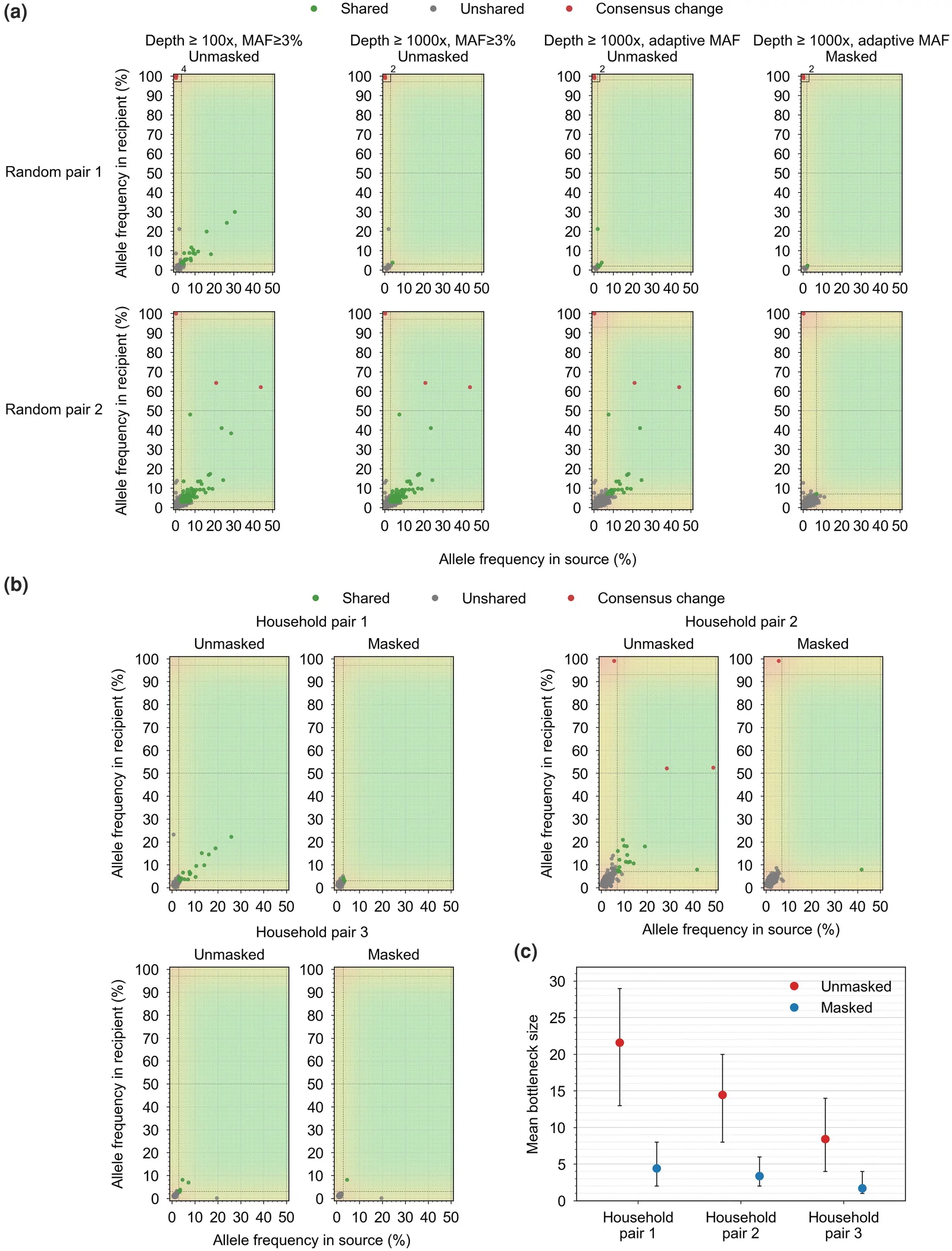
iSNV frequencies in different pairs of samples and estimated bottleneck sizes before and after masking. a) iSNV frequencies in two randomly paired samples processed at the same sequencing center. The samples on the *x* axis were sampled approximately 1 week before the samples on the *y* axis, but the samples within each pair are from different households and therefore unlikely to be direct transmission pairs. Each column of plots shows variant frequencies under different criteria, using various depth and MAF thresholds to call iSNVs and with (rightmost column) or without masking highly shared, artifactual positions. Three categories of points show minor variants that are shared between the source-recipient pair, variants where the consensus differs between the two samples, and minor variants that are below the analysis MAF threshold used for the given sequencing center and method in either the source or recipient. The number in the top-left corner of the top row of plots gives the number of variants that are all below the MAF threshold in the first samples and close to 100% frequency in the second sample (full SNP). b) iSNV frequencies in three epidemiologically linked samples, where one household member (source) tested positive approximately 1 week before the other member (recipient). Plots on the left side show variants before masking artifacts, while plots on the right show the variants remaining after masking problematic artifact positions using the adaptive masking scheme. Here, both unmasked and masked plots show variants called under a 1,000× depth and the analysis MAF threshold for the given sequencing center and method. Variants are classified as in (a). c) Estimated bottleneck size estimates for each household pair from (b) before and after masking artifactual sites. Points indicate the posterior mean bottleneck estimate for unmasked and masked data, as indicated in the legend, and error bars indicate the 95% highest posterior density intervals.

As a negative control, we first analyzed randomly selected, nonhousehold sample pairs from the same sequencing centers (Fig. 4a). Prior to masking, several apparently shared variants were observed between unrelated samples, particularly at lower read-depth and MAF thresholds. In the absence of epidemiological context, such patterns could be misinterpreted as evidence of transmission. However, as read-depth thresholds were increased and MAF thresholds adapted to the overall noisiness of the data, these apparent similarities diminished. After masking highly shared variants using the adaptive masking scheme, no convincing shared within-host diversity remained, leaving only fixed consensus differences and low-frequency noise below the analysis MAF thresholds.

We next examined likely household transmission pairs from the ONS-CIS, focusing on households in which one individual tested positive at least 1 week before any other member, with subsequent household member(s) testing positive 7 to 10 days later, suggesting direct transmission and a clear index case. For three such pairs, we compared iSNV frequencies and fixed consensus changes between source and recipient samples before and after masking artifactual positions using the adaptive masking scheme (Fig. 4b and c). The transmission bottleneck size was estimated using the beta-binomial method (Sobel Leonard et al. 2017) but adapted to use a Bayesian Markov Chain Monte Carlo (MCMC) approach instead of the original maximum-likelihood framework, allowing the presentation of highest posterior density intervals for the bottleneck size instead of the likelihood ratio test estimates of the original manuscript (see Methods). In the first pair, unmasked data showed multiple shared iSNVs at similar frequencies in source and recipient (Fig. 4b, household pair 1), suggesting transmission of a genetically diverse viral population and yielding a large inferred transmission bottleneck (mean bottleneck estimate exceeding 20 virions) (Fig. 4c). After masking, most of this apparent shared diversity disappeared, leaving only a small number of shared variants just above the analysis MAF threshold and resulting in a substantially reduced bottleneck estimate. In the second pair, the unmasked data again showed numerous shared variants across a wide frequency range, most of which were removed by masking. However, one shared iSNV and another variant that was just below the analysis MAF threshold in the source but fixed in the recipient persisted, still supporting a direct transmission link between the samples (Fig. 4b). The third pair showed a few low-frequency shared variants, with only one remaining after masking. Across all three example pairs, masking substantially reduced the estimated bottleneck sizes, yielding smaller and more consistent estimates of approximately two to five virions. These narrower estimates imply that only a few virions typically initiated infection in the recipient, aligning with the expectation of tight transmission bottlenecks for SARS-CoV-2 and consistent with previous reports (Lythgoe et al. 2021; Hannon et al. 2022; Bendall et al. 2023).

## Discussion

Analyses of within-host viral variation inferred from deep-sequencing data are increasingly used to study viral evolution and transmission dynamics. For SARS-CoV-2, which has been sequenced at an exceptional scale across diverse laboratories and protocols, such analyses place particularly strong demands on the reliability of low-frequency variant detection. iSNVs have been used to quantify within-host diversity, investigate putative transmission links, and estimate transmission bottleneck sizes (Popa et al. 2020; Lythgoe et al. 2021; Martin and Koelle 2021; Tonkin-Hill et al. 2021; Hannon et al. 2022; Bendall et al. 2023). These applications offer insights that are not accessible from consensus genomes alone, but they also critically rely on the assumption that the low-frequency variants predominantly reflect true biological signal rather than technical artifacts.

However, we and others have observed iSNVs that appear in the data more frequently than is biologically plausible, with some highly shared variants persisting even when conservative MAF thresholds are applied (Lythgoe et al. 2021; Martin and Koelle 2021; Walter et al. 2023; Mostefai et al. 2024). Such recurrence raises concerns about the reliability of iSNV-based inference when systematic technical artifacts are not appropriately accounted for. Previous efforts to control likely artifactual iSNVs have largely relied on ad hoc, study-specific filtering strategies. These include restricting analyses to variants supported across technical or biological replicates (McCrone and Lauring 2016; Grubaugh et al. 2019; Braun et al. 2021; Tonkin-Hill et al. 2021), removing variants that are shared across a large proportion of samples within a dataset (Lythgoe et al. 2021), and excluding sites flagged as problematic in consensus-level analyses aimed at identifying lab-associated or recurrent error in global datasets (De Maio et al. 2020; Turakhia et al. 2020). While these approaches can remove well-characterized error modes, they are typically applied as fixed rules and do not explicitly account for variation in noise structure across datasets.

Here, we developed a systematic and dataset-aware framework for identifying and masking recurrent artifactual iSNVs. Rather than assuming a universal frequency threshold or relying on predefined problematic-site lists, our approach exploits recurrence patterns observed across samples within each sequencing dataset to identify variants that are unlikely to reflect genuine within-host variation. The framework yields small, targeted sets of positions to mask and is designed to be scalable to diverse, large-scale sequencing datasets, while explicitly accommodating heterogeneity in noise across different sequencing centers and sample processing protocols.

Applying this framework to data from the ONS-CIS showed that recurrent artifacts are predominantly sequencing center-specific rather than primer-specific. Each sequencing center-method combination exhibited a modest but distinct set of recurrent artifactual variants, typically numbering only a few dozen sites, with limited overlap between centers and minimal overlap with widely used consensus-level problematic-site lists (De Maio et al. 2020). This pattern indicates that systematic artifacts arise primarily from local laboratory- or batch-associated processes and protocols rather than from the primer sets themselves. Similar center-specific structure has been observed in another large-scale analysis of public SARS-CoV-2 NGS libraries, where data-driven approaches, including dimensionality reduction, were used to distinguish systematic artifacts from putative within-host mutations (Mostefai et al. 2024). More generally, while model-based variant quality frameworks integrating multiple sources of technical bias are widely used in human genomics (eg GATK; Van der Auwera et al. 2013), analogous approaches tailored to viral deep-sequencing data remain largely unexplored.

Although here we focused on SARS-CoV-2 in humans, and specifically data from the ONS-CIS, other pathogen systems might be expected to be subject to artifactual variants. At the sub-consensus level, artifactual variants have been observed in a large number of studies, including other viruses (Worby et al. 2017), bacteria (Senghore et al. 2023), and human DNA (Meacham et al. 2011). The framework we applied to SARS-CoV-2 among the ONS-CIS samples relied on knowing the expected number of iSNVs per sample, but this is unlikely to be known in other systems. The alternative prevalent mask scheme we suggested does not rely on this expectation and could therefore provide a good starting point where there is less prior knowledge and provide a systematic way to interrogate the data. Nonetheless, whatever scheme is used, analysts should be mindful that the different biological and technical processes that generate variants in NGS data will produce different patterns, and therefore, the masking scheme and MAF and sharing thresholds used will inevitably affect which variants are identified. Fundamentally, it is unlikely there will be a one-fits-all strategy that will work across all systems.

The identification and masking of artifactual iSNVs remain inherently constrained by trade-offs between sensitivity and specificity that apply to within-host analyses more generally. Even under stringent quality assurance, low-frequency artifacts can persist, and the distinction between biological signal and noise depends on how within-host variation is measured and summarized. In settings where genuine within-host evolution is expected, such as persistent infections in immunocompromised individuals, replicate sequencing can help validate minor variants (Ben Zvi et al. 2025), but such approaches are rarely feasible at scale, do not generalize to routine sequencing datasets, and may fail to identify very highly shared artifacts as problematic since they may appear in both replicates. More broadly, measures of within-host diversity remain sensitive to sequencing depth, sampling variance, and detection thresholds, which can bias diversity estimates and complicate comparisons across samples or studies if not carefully considered (Zhao and Illingworth 2019; Lauring 2020).

Failure to identify and account for recurrent artifactual iSNVs can substantially distort biological inference based on within-host variation. Artifactual variants can inflate estimates of within-host diversity, generate spurious genetic similarity between unrelated samples, and bias transmission bottleneck estimates toward unrealistically large values. Shared low-frequency variants, when interpreted without appropriate within-dataset controls, may therefore be mistaken for evidence of direct transmission, even though such variants are often observed between epidemiologically unrelated samples (Walter et al. 2023). These methodological effects may contribute to the wide range of SARS-CoV-2 transmission bottleneck estimates reported across studies (Popa et al. 2020; Lythgoe et al. 2021; Hannon et al. 2022; Bendall et al. 2023), alongside genuine biological and epidemiological variation.

Overall, these findings show that recurrent artifactual iSNVs are common, often sequencing center-specific, and capable of strongly biasing within-host and transmission-level inference if left unaddressed. Reliable use of iSNV data therefore requires systematic, dataset-aware artifact control rather than reliance on fixed thresholds or universal masking rules. As genomic surveillance increasingly seeks to exploit sub-consensus variation, transparent recurrence-aware strategies, together with comprehensive and standardized recording of laboratory and sequencing metadata in large sequence databases, will be essential to ensure that evolutionary and epidemiological conclusions reflect viral biology rather than technical noise for SARS-CoV-2 and other pathogens and for future pathogen surveillance efforts.

## Materials and methods

### ONS COVID-19 infection survey

The ONS-CIS was a UK household-based surveillance study that ran between 2020 April 26 and 2023 March 31 before being paused and then closed (Lythgoe et al. 2021; Lythgoe et al. 2023). During this period, more than 225,000 households were recruited on a rolling basis from across the United Kingdom with the aim of being as representative as possible. Written consent was obtained from all participants, and all consenting individuals aged 2 years and older from each household provided weekly nose + throat swab samples for the first 4 weeks after recruitment and monthly swabs thereafter. All swabs were tested for SARS-CoV-2 by RT-PCR. Between April 2020 and November 2020, a selection of RT-PCR-positive samples were sequenced each week, with some additional retrospective sequencing of stored samples. From December 2020 onwards, all samples with a Ct ≤30 were sent to one of several participating laboratories for sequencing.

### Library preparation and sequencing

All samples analyzed in this study were sequenced on Illumina platforms (MiSeq, NovaSeq, or NextSeq). Two major sequencing approaches were used: the ARTIC amplicon protocol (with primer versions 3, 4.0, and 4.1), with consensus FASTA sequence files generated using the ARTIC nextflow processing pipeline (Davis James et al. 2021; Ulhuq et al. 2023), or ve-SEQ, an RNASeq protocol based on a quantitative targeted enrichment strategy (Bonsall et al. 2015; Bonsall et al. 2020; Lythgoe et al. 2021), with consensus sequences and BAM files produced using Shiver (Wymant et al. 2018). For ARTIC datasets, library preparation methods varied by sequencing center (Table 1) and included Illumina DNA Prep (previously Nextera DNA Flex), Nextera XT, and NEBNext Ultra II-based workflows. At Northumbria University (NORT) and Quadram Institute Bioscience (NORW), libraries were prepared using the CoronaHiT method (Baker et al. 2021). Some other samples were generated using the ARTIC LoCost protocol (Quick 2020); however, the specific library preparation workflows were not consistently recorded in the available metadata.

Eight originating laboratories participated, and samples were sequenced at five different sequencing laboratories. Samples are referred to these sequencing laboratories throughout this paper according to the short codes: NORT, NORW, OXON, PHEC, and SANG (Table 1).

### Bioinformatic processing

The processing of OXON sequence data (ve-SEQ protocol) has been previously described (Lythgoe et al. 2021). Briefly, sequences were preprocessed by downloading FASTQ files from CLIMB-COVID (Nicholls et al. 2021) and classifying them using Kraken 2 (Wood et al. 2019) with a custom database including the human genome (GRCh38) and the full bacterial/viral RefSeq set downloaded in May 2020. Human and bacterial sequences were filtered out using Castanet's filter_keep_reads.py (Goh et al. 2019). The remaining viral and unclassified reads were trimmed to remove primers and adapters using Trimmomatic (Bolger et al. 2014) v0.36. These reads were mapped to the SARS-CoV-2 RefSeq genome (Wuhan-Hu-1 isolate MN908947.3) using shiver_map_reads.sh from the Shiver (Wymant et al. 2018) v1.5.7 package and Smalt (https://github.com/rcallahan/smalt) as the read mapper. Shiver provides base frequency (BaseFreq) files, which are .csv files containing the frequencies of A, C, G, T nucleotides, gaps, and ambiguities for each position in the genome. All genomes were already aligned to Wuhan-Hu-1 following processing with Shiver, so no further alignment was required. Consensus sequences were constructed using an in-house Python script that takes each BaseFreq and calculates the most common nucleotide or gap or “N” at that position using a 60% majority and a read depth of 5×. If there were fewer than five reads at a position, “N” was called. Lineages were determined using Pangolin v4.3.132 (O’Toole et al. 2021).

For all other sequencing centers, raw sequencing data were, to our knowledge, generally processed using https://github.com/connor-lab/ncov2019-artic-nf, a Nextflow implementation of the ARTIC Network fieldbioinformatics pipeline, which generated consensus sequences and intermediate BAM files. For our analysis, we downloaded BAM files from CLIMB as provided by the sequencing centers, which we then processed with Shiver to produce BaseFreq files. Minimal additional filtering was applied before BaseFreq generation: unmapped reads and supplementary alignments were removed, improper or incompletely mapped read pairs were excluded for paired-end data, and only bases with Phred quality ≥5 were counted in the pileup.

Only samples that passed a genome coverage quality filter of a minimum of ten non-gap reads across ≥50% of the genome (defined as ≥14,000 bases) were included in our analyses.

### Identifying iSNVs

We used a custom Python script to identify iSNVs within each SARS-CoV-2 genome, excluding positions 1 to 265 at the 5′ end and 29,675 to 29,903 at the 3′ end, the latter encompassing the poly-A tail. An iSNV was called if the frequency of the most common minor allele at a position in the genome was above a specific threshold (ranging between 2% and 20%), provided there was a total sequencing depth of at least 1,000× (excluding gaps) at that position. Positions with a gap or ambiguity as the majority allele were excluded from analysis, and minor allele gaps were not considered as iSNVs. Initially, we also analyzed iSNVs called at positions with minimum depth thresholds of 10× or 100× total reads under the various MAF thresholds to compare with the stricter 1,000×, depth threshold.

For each sequencing center, the proportion of samples with an iSNV at each genomic position was determined, and the data were further subdivided by ARTIC primer version if multiple primer sets were used by that center. The combination of sequencing approach and primer set, where applicable, is referred to as the sequencing method. MAF thresholds for calling iSNVs ranged from 2% to 20% by increments of 1%.

### Identification of highly shared iSNVs and potential masking sets

To consider both sequencing noise and artifactual, highly shared iSNVs in the data, we analyzed the distribution and sharedness of iSNVs across genomic positions using various MAF thresholds and thresholds for the allowed sharedness of iSNVs among samples from the same sequencing center regardless of method and from the same sequencing center-method combination. Specifically, for each MAF threshold between 2% and 20% (in 1% increments), we analyzed the total number of iSNVs per sample as well as the total number of samples from the group of samples we were considering with an iSNV at each genomic position.

To get schemes of possible masking sets of artifactual iSNVs among samples from the same sequencing center and method, we varied thresholds of allowed sharedness of iSNVs among samples from >0.5% to >20% in 0.5% increments, given the varied MAF thresholds for calling iSNVs. For each combination of MAF and sharedness thresholds, we identified a set of positions where iSNVs were called “too frequently” and thus considered these artifact positions to mask. For example, if the MAF threshold was set to 2% and the sharedness threshold at 1.0%, every position where more than 1% of samples from a given sequencing center-method category had a minor variant with a frequency of 2% or more was added to the masking set. This resulted in up to 760 different possible masking schemes for each sequencing center-method combination.

### Choosing masking sets and MAF thresholds for true versus artifactual iSNVs

To determine a set of masking positions for each sequencing center-method combination that considered both noise and sequencing artifacts while preserving the majority of true iSNVs, we constructed adaptive masks using the OXON dataset as a reference (Lythgoe et al. 2021). Using the mean number of iSNVs per sample as a metric for noise in the data, we identified the MAF thresholds at which data from each sequencing center and method had a mean number of iSNVs per sample closest to the number observed for samples processed with the OXON method (∼10 iSNVs). This MAF threshold was then used for masking positions. Specifically, following Lythgoe et al. (2021), we used a 1.5% threshold for the proportion of samples (from the same sequencing center-method combination) sharing the same iSNV positions to call an artifactual position. After masking the identified positions, samples were again analyzed for their average number of iSNVs, and an “analysis” MAF threshold for each dataset was proposed where the masked data were now closest to having the reference average number of iSNVs per sample.

As an alternative masking strategy, sets were also compiled from the most commonly masked positions across all tested MAF and sharedness threshold combinations for each sequencing center-method combination. For these sets, every position that was present in >20% of possible masking schemes for a sequencing center-method combination was considered artifactual and masked. Using this scheme (the prevalent mask), analysis MAF thresholds for subsequent analysis could again be determined with the OXON data as a reference, aiming for an average number of iSNVs per sample close to 10.

### Analyzing strand bias across a subset of samples from each sequencing center

We analyzed strand bias across a random subset of 100 samples per sequencing center-method combination to identify positions where the distribution of minor variant reads between forward and reverse strands was systematically biased. Samples from the OXON sequencing center were excluded because the ve-SEQ protocol (capture-based) is not prone to the same primer-related strand artifacts as ARTIC amplicon sequencing. Variant calling was performed with LoFreq (Wilm et al. 2012) (v2.1.5) against the SARS-CoV-2 reference genome (MN908947.3) with default post-filters disabled (–no-default-filter). We parsed the VCF output per sample, extracting strand-specific counts from the INFO column DP4 (ref-forward, ref-reverse, alt-forward, alt-reverse) and allele frequencies from AF.

For each sequencing center-method combination, at each genomic position between 265 and 29,674, we pooled strand-specific counts across the subsampled set, provided at least ten samples contributed data and the pooled depth was ≥1,000 reads. The major versus minor allele was defined per sample using AF (if AF >0.5, the alternative allele, ALT, is major; otherwise, the reference, REF, is major). When multiple ALT <0.5 were present, the one with the most reads was used as the minor allele. We then tested a 2 × 2 table (major/minor × forward/reverse) using a two-sided Fisher's exact test. Positions were excluded if underpowered: <10 contributing samples, pooled depth <1,000 reads, any zero cell or expected cell count <5 in the 2 × 2 table, or sensitive to a small pseudo-count (adding 0.5 to all cells changed the *P*-value by >0.1). Raw *P*-values were adjusted across positions using the Benjamini–Hochberg procedure, and finally positions with *q* < 0.01 were designated as strand-biased for that sequencing center-method combination and used in downstream comparisons with other mask sets.

### Comparison between masking sets and known problematic positions

To characterize and analyze possible explanations for the sets of masked iSNVs in each sequencing center, we compared each masking set to each other and to sets of potentially problematic sites. These included (i) known problematic positions (De Maio Mask and De Maio Caution) (De Maio et al. 2020), (ii) primer-binding positions from ARTIC primer schemes v3, v4, and v4.1, and (iii) positions with significant strand bias identified across subsets of samples from each sequencing center. Overlaps and unique positions among these sets were visualized using UpSet plots generated with the UpSetR R package (Conway et al. 2017).

### Test of enrichment of masked sites in primer-binding regions

For each ARTIC primer version (v3, v4, and v4.1), we tested whether masked sites occurred within primer-binding regions more often than expected by chance. For each version, we formed the union of unique masked genomic positions across masking sets using that primer scheme (eg NORT ARTIC 3, PHEC ARTIC 3, SANG ARTIC 3). Primer-binding coordinates were taken from the corresponding ARTIC scheme (downloaded from https://github.com/artic-network/primer-schemes/tree/master/nCoV-2019). Using the full SARS-CoV-2 genome as background (*N* = 29,903), we let *K* be the number of primer positions, *M* the number of masked positions in the union, and *x* the observed overlap. Under a null of uniform placement without replacement, *X* ∼ Hypergeom(*N*,*K*,*M*). One-sided enrichment *P*-values were computed as *p* = [*X* ≥ *x*], using phyper in R. We also reported primer coverage *K*/*N*, the expected overlaps *M* × *K*/*N*, and fold enrichment (observed/expected). Because three tests were prespecified (one per primer version), *P*-values were Benjamini–Hochberg adjusted and reported as *q*-values.

### Bottleneck size calculation

The minor allele frequencies from three likely transmission pairs were analyzed using a modified version of the exact beta-binomial method of Sobel Leonard et al. (2017). In this study, sites were considered as potential iSNVs only if the depth at that position was ≥1,000 reads in the source individual and the iSNV frequency at that position was above the analysis MAF threshold for the source's sequencing center.

A Bayesian MCMC approach was used instead of the original maximum-likelihood framework, allowing the presentation of highest posterior density intervals for the bottleneck size instead of the likelihood ratio test estimates of the original manuscript. The MCMC approach requires a prior distribution for the size of the bottleneck, which in this case was a (shifted) geometric distribution with a success probability of 0.5, resulting in a mean of 2 virions. Each MCMC chain was run for 50,000 iterations, with the first 5,000 discarded as burn-in. This process was repeated twice for each pair, first without masking any genome positions and then with masking using the adaptive mask scheme. If the source and recipients of the pair came from different sequencing centers, all positions from both mask lists were masked. The variant calling threshold in the recipient was set to the analysis threshold for the recipient's sequencing center.

### Notes

This work contains statistical data from ONS, which is Crown Copyright. The use of the ONS statistical data in this work does not imply the endorsement of the ONS in relation to the interpretation or analysis of the statistical data. This work uses research datasets that may not exactly reproduce National Statistics aggregates.

## Supplementary Material

msag209_Supplementary_Data

## Data Availability

All custom code written and described in this manuscript are available at https://github.com/lythgoe-lab/SC2_artefacts. All sequences are publicly available via the COG-UK project on the ENA (https://www.ebi.ac.uk/ena/browser/view/PRJEB37886).
